# Biomarkers of Treatment Response in Paediatric Medulloblastoma

**DOI:** 10.3390/diagnostics16071089

**Published:** 2026-04-03

**Authors:** Mirgul Bayanova, Timur Saliev, Askhat Zhakupov, Aizhan Abdikadirova, Malika Sapargaliyeva, Bakytkali Ibraimov, Aidos Bolatov

**Affiliations:** 1 Department of Clinical and Genetic Diagnostics, University Medical Center, Corporate Fund, Astana 010000, Kazakhstan; 2 Institute for Fundamental and Applied Medical Research, S.D. Asfendiyarov Kazakh National Medical University, Almaty 050000, Kazakhstan; 3 Department of Pathology, University Medical Center, Corporate Fund, Astana 010000, Kazakhstan; 4 Shenzhen University Medical School, Shenzhen University, Shenzhen 518060, China

**Keywords:** paediatric medulloblastoma, protein biomarkers, brain tumour, imaging, WNT, sonic hedgehog

## Abstract

Paediatric medulloblastoma is the most common malignant brain tumour in children, exhibiting substantial biological heterogeneity that drives variable treatment outcomes. Despite advances in multimodal therapy, treatment-related morbidity remains a critical concern, underscoring the need for biomarkers to guide precision therapy. This review synthesises current knowledge on biomarkers of treatment response, encompassing molecular, epigenetic, transcriptomic, protein, and imaging-based markers. WNT-activated tumours show excellent prognosis and are candidates for therapy de-escalation; SHH-driven tumours demonstrate age-dependent outcomes influenced by TP53 status; Group 3 tumours carry the poorest prognosis; and Group 4 tumours display highly variable outcomes. DNA methylation profiles, transcriptional programs, and non-coding RNAs provide additional predictive insights. Protein biomarkers and advanced imaging, including liquid biopsy and radiomics, offer minimally invasive approaches for real-time monitoring of treatment efficacy. The review also addresses challenges such as intra-tumour heterogeneity, limited tissue availability, technical variability, and ethical considerations in paediatric oncology. Finally, we explore future directions, highlighting integrative, longitudinal, and ethically grounded biomarker strategies that have the potential to optimise therapy, minimise long-term toxicity, and improve both survival and quality of life for children with medulloblastoma.

## 1. Introduction

Medulloblastoma, the most common malignant brain tumour in children, originates in the cerebellum and is a leading cause of cancer-related death and long-term disability in paediatric oncology [1]. Advances in neurosurgery, radiotherapy, and chemotherapy have improved survival, but at the cost of significant treatment-related toxicity, including neurocognitive impairment, endocrine dysfunction, and hearing loss [2]. As survival rates improve, optimising efficacy while minimising harm has become a central goal [3].

Outcomes remain highly variable despite uniform treatment paradigms based largely on clinical and radiological risk stratification [4]. Standard approaches fail to account for the underlying biological diversity of the disease: some patients achieve durable remission with modest treatment intensity, whereas others relapse early despite aggressive therapy [5,6,7]. Improved understanding of the molecular landscape has revealed that medulloblastoma comprises biologically distinct entities rather than a single pathological diagnosis [6,8,9].

A wide array of candidate biomarkers has been investigated in paediatric medulloblastoma, encompassing genetic alterations, epigenetic modifications, transcriptomic signatures, protein expression patterns, and advanced imaging features [10]. Nevertheless, the translation of biomarker discoveries into routine clinical practice remains uneven. While molecular subgrouping has become increasingly embedded in diagnostic workflows and clinical trial design, many proposed biomarkers of treatment response lack prospective validation and standardisation. Moreover, the ethical and logistical complexities of paediatric oncology, including limited tissue availability and the imperative to minimise harm, pose additional challenges to biomarker-driven approaches [11].

Clinically, medulloblastoma typically presents with symptoms of increased intracranial pressure (headache, vomiting) and cerebellar dysfunction (truncal ataxia, dysmetria), often progressing rapidly over weeks to months. Historically, diagnosis has relied on histopathological examination of tumour tissue, which classically reveals small, round, blue-cell tumors with high cellularity, frequent mitoses, and a propensity for neuronal differentiation, classically forming Homer-Wright rosettes. However, these histological features alone are insufficient to predict the highly variable clinical outcomes observed, underscoring the need for the molecular and biomarker-driven approaches detailed in this review.

The article aims to synthesise current knowledge on biomarkers of treatment response in paediatric medulloblastoma, with a particular focus on molecular, epigenetic, transcriptomic, protein, and imaging-based markers.

## 2. Molecular Classification and Predictive Biomarkers

Four principal molecular subgroups, WNT, Sonic Hedgehog (SHH), Group 3, and Group 4, are defined by characteristic genetic, epigenetic, and transcriptional profiles that function as predictive biomarkers of treatment response [12]. However, classification methods (immunohistochemistry, DNA methylation arrays, gene expression profiling) vary in diagnostic capability, cost, and accessibility, influencing result reproducibility across settings [12].

WNT-activated medulloblastomas, accounting for approximately 10% of paediatric cases, are characterised by aberrant activation of the WNT/β-catenin signalling pathway, most commonly through activating mutations in the CTNNB1 gene, along with monosomy 6 and highly specific DNA methylation signatures [4] (Table 1). WNT tumours exhibit low metastatic potential, with fewer than 10% of patients presenting with metastatic disease at diagnosis. These tumours consistently demonstrate remarkable sensitivity to standard multimodal therapy, with long-term survival rates exceeding 90% [13]. Biomarkers such as nuclear β-catenin accumulation and subgroup-specific methylation patterns reliably predict a favourable response and low metastatic potential. The robustness of these biomarkers has enabled clinical trials investigating therapy de-escalation strategies aimed at reducing craniospinal irradiation doses and long-term neurotoxicity without compromising survival [14]. Several key studies collectively illustrate this progress, offering both practical solutions for clinical settings and deeper insights into tumour biology.

Recent work has expanded our diagnostic and biological understanding: Ranade et al. (2025) demonstrated that diffuse immunohistochemical staining for lymphoid enhancer-binding factor 1 (LEF1) achieves 100% specificity for WNT-activated tumours, offering a cost-effective diagnostic tool for resource-limited settings [15].

Additionally, Alaña et al. (2022) identified a novel CTNNB1 in-frame deletion (p.Ser37del) with gain-of-function properties, expanding the known mutational landscape [16]. Importantly, Moreno et al. (2023) reported that 27% of WNT-activated tumours in a Latin-Iberian cohort were CTNNB1 wild-type, many with germline APC mutations, suggesting a higher hereditary component in some populations [17]. The robustness of WNT biomarkers has enabled clinical trials investigating therapy de-escalation strategies aimed at reducing craniospinal irradiation doses and long-term neurotoxicity without compromising survival [18].

Beyond diagnosis and genetics, recent work is also exploring new therapeutic vulnerabilities. Marques et al. (2023) provided a comprehensive immune checkpoint profile of medulloblastoma, revealing a striking absence of mRNA expression for canonical immunotherapy targets like PD-1, PD-L1, and CTLA-4 [19]. Instead, they identified significant overexpression of CD24 and CD276 (B7-H3) across molecular subgroups, with high levels correlating with worse survival. This pivotal shift in the immunogenic profile suggests that future immunotherapeutic strategies should pivot away from traditional checkpoint inhibitors and instead investigate CD24 and CD276 as promising novel targets.

SHH medulloblastomas constitute approximately one quarter to one third of paediatric cases and display substantial biological heterogeneity driven by dysregulation of the Hedgehog signalling pathway [20] (Table 1). Metastatic dissemination occurs in approximately 15–20% of SHH medulloblastomas, with higher frequencies observed in TP53-mutant and older patient populations. Recurrent alterations include mutations in PTCH1, SMO, SUFU, and amplification of GLI transcription factors [21]. Importantly, predictive biomarkers within the SHH subgroup are strongly influenced by patient age and tumour genetics. TP53 mutation status has emerged as one of the most powerful predictors of treatment response, with TP53-mutant SHH tumours exhibiting marked genomic instability, resistance to DNA-damaging therapies, and poor clinical outcomes [22]. In contrast, TP53–wild-type SHH tumours, particularly in infants, often respond favourably to chemotherapy-based regimens that minimise or omit radiotherapy [23]. Although targeted inhibition of the SHH pathway using SMO inhibitors has demonstrated clinical activity in selected cases, the frequent emergence of resistance underscores the need for biomarkers capable of predicting durable therapeutic response.

Group 3 medulloblastomas represent the most aggressive molecular subtype and are associated with the poorest prognosis [24] (Table 1). These tumours are characterised by the highest metastatic propensity among all subgroups, with 40–45% of patients presenting with disseminated disease at diagnosis. Metastatic status in Group 3 is strongly associated with MYC amplification and confers a particularly poor prognosis. These tumours are frequently characterised by MYC amplification or overexpression, extensive chromosomal instability, and a high incidence of metastatic disease at diagnosis. MYC status functions as a strong predictive biomarker of treatment resistance and early relapse, identifying patients at the highest risk for treatment failure under standard protocols [25]. Additional transcriptional signatures linked to stemness, hypoxia, and immune evasion further correlate with poor therapeutic response, suggesting that both intrinsic tumour biology and the tumour microenvironment contribute to resistance. These biomarker profiles have prompted investigation of intensified treatment regimens and novel therapeutic strategies, including targeted agents and immunotherapies, for this high-risk population.

**Table 1:** Summary of the molecular classification and predictive biomarkers of medulloblastoma.

Molecular Subgroup	Frequency	Key Molecular Features/Genetic Alterations	Predictive Biomarkers	Clinical Characteristics/Prognosis	Therapeutic Implications	Metastatic Potential	Ref.
WNT	~10%	Activation of WNT/β-catenin pathway; CTNNB1 mutations; monosomy 6; specific DNA methylation signatures	Nuclear β-catenin accumulation; subgroup-specific methylation patterns	Excellent prognosis; low metastatic potential	High sensitivity to standard therapy; potential for therapy de-escalation to reduce craniospinal irradiation and long-term neurotoxicity	Low (<10% metastatic at diagnosis)	[26,27]
SHH	~25–33%	Dysregulation of Hedgehog pathway; mutations in PTCH1, SMO, SUFU; GLI amplification	TP53 status (mutant vs. wild-type); age and Tumour genetics influence biomarker relevance	Heterogeneous outcomes; TP53-mutant Tumours show poor prognosis; TP53–wild-type Tumours (especially infants) respond better	Chemotherapy-based regimens preferred for some patients; targeted SMO inhibitors show activity but resistance common; biomarkers guide individualized therapy	Moderate (15–20% metastatic)	[28,29]
Group 3	~15–20% (approx.)	MYC amplification/overexpression; extensive chromosomal instability; high metastasis at diagnosis	MYC status; transcriptional signatures linked to stemness, hypoxia, immune evasion	Most aggressive subtype; poorest prognosis; early relapse common	High-risk patients may need intensified therapy, novel targeted agents, or immunotherapies	High (40–45% metastatic)	[30,31]
Group 4	~35–40%	Isochromosome 17q; SNCAIP duplication; enhancer hijacking of GFI1, PRDM6; heterogeneous genomic profiles	DNA methylation patterns; copy number variation profiles emerging	Largest and most heterogeneous subgroup; intermediate, variable outcomes	Biomarker-driven risk stratification emerging; may guide individualized therapy	Moderate (30–35% metastatic; lower in subtypes 4/7)	[32,33]

Group 4 medulloblastomas comprise the largest and most heterogeneous subgroup, accounting for approximately 35–40% of cases, with intermediate but highly variable clinical outcomes [34]. Unlike other subgroups, Group 4 tumours lack a dominant oncogenic driver, complicating biomarker development. Common molecular features include isochromosome 17q, SNCAIP duplication, and enhancer hijacking events involving oncogenes such as GFI1 and PRDM6 [35]. Emerging technologies such as optical genome mapping (OGM) are further refining our understanding of the genomic landscape in these subgroups by enabling high-resolution, genome-wide detection of structural variants (SVs). A recent study employing OGM identified novel genomic amplifications, including one affecting the GPC5 gene in a Group 3 tumour, and discovered recurrent SVs impacting the NRXN1 gene in subsets of both Group 3 and Group 4 medulloblastomas, highlighting previously underappreciated complexity [36].

Metastatic dissemination occurs in 30–35% of Group 4 patients, though outcomes are highly variable and influenced by molecular substructure; subtypes 4 and 7 show lower metastatic risk compared to other Group 4 variants.

Recent integrative genomic and epigenomic analyses have revealed biologically meaningful substructures within Group 4 that correlate with metastatic propensity and treatment response. Specifically, DNA methylation profiling has identified distinct subtypes, including subtypes 4 and 7, that carry prognostic significance; subtype 7 is associated with more favourable outcomes and greater treatment sensitivity, while other subtypes confer a higher risk of relapse. In this context, DNA methylation patterns and copy number variation profiles are emerging as promising predictive biomarkers that may enable more refined risk stratification and individualised therapy.

## 3. Epigenetic and Transcriptomic Biomarkers

Epigenetic and transcriptomic biomarkers have emerged as critical determinants of treatment response in paediatric medulloblastoma, providing insights into tumour biology that extend beyond static genetic alterations [37]. These biomarkers reflect dynamic regulatory processes that govern gene expression, cellular differentiation, and adaptive responses to therapy, thereby offering powerful tools for both tumour classification and prediction of therapeutic sensitivity or resistance. Among epigenetic mechanisms, DNA methylation has proven particularly informative, while transcriptomic profiling has revealed gene expression programmes associated with tumour aggressiveness, metastatic potential, and treatment failure [38].

DNA methylation profiling is now considered one of the most robust and clinically actionable approaches for medulloblastoma classification (Table 2). Methylation-based tumour stratification reliably distinguishes the four major molecular subgroups and their emerging substructures with high accuracy, even in cases with limited or ambiguous histopathological features [39]. Importantly, specific methylation signatures have been shown to correlate with treatment response and clinical outcome, particularly within Group 3 and Group 4 tumours, where traditional genetic drivers are less clearly defined. Aberrant methylation of genes involved in cell cycle regulation, DNA repair, and neuronal differentiation has been associated with resistance to chemotherapy and radiotherapy, whereas methylation patterns reflecting more differentiated cellular states tend to correlate with improved treatment sensitivity [40]. The relative stability of DNA methylation profiles, combined with their strong prognostic relevance, has facilitated their incorporation into diagnostic workflows and clinical trials.

Recent studies employing advanced molecular and proteomic techniques are refining the risk stratification and clinical management of medulloblastoma, moving beyond the four core subgroups to identify finer prognostic distinctions and more practical biomarkers. These investigations collectively highlight the growing importance of integrating protein-level data and detailed methylation profiling into clinical decision-making to better predict outcomes and tailor therapies.

A pivotal contribution comes from the high-resolution proteomic analysis by Delaidelli et al. (2025) [8], which identified MYC protein expression, detectable by routine immunohistochemistry (IHC), as a powerful independent predictor of therapy resistance and poor survival in non-WNT medulloblastomas. Notably, this MYC-driven proteomic signature was present in only about half of the IHC-positive tumours that harboured MYC amplification, indicating that protein overexpression itself is a critical biological driver. The study convincingly argues that integrating MYC IHC into clinical algorithms could improve global risk stratification, reclassifying approximately 20% of patients into a very high-risk category, independent of complex molecular testing.

Further granularity within established subgroups is revealed through DNA methylation profiling, particularly for the sonic hedgehog (SHH) subtype. Tonn et al. (2023) demonstrated significant heterogeneity within early childhood SHH medulloblastomas treated with radiation-avoiding chemotherapy [41]. Their methylation analysis subdivided the SHH-2 category into two clinically distinct entities: a very low-risk group (SHH-2a), enriched for MBEN histology and SMO mutations, and a higher-risk group (SHH-2b), consisting of older patients with desmoplastic histology and a greater relapse risk. This work underscores that methylation-based subclassification can uncover prognostically relevant biological diversity that is not apparent from histology or core molecular subgrouping alone, directly impacting treatment intensity decisions for young children.

The clinical utility of this refined molecular stratification is also supported by evidence from challenging metastatic cases. Irikura et al. (2023) reported on long-term survivors of metastatic medulloblastoma who were successfully treated with a reduced dose of craniospinal irradiation (24 Gy) [42]. DNA methylation analysis revealed that these patients belonged to specific, more favorable subtypes within the Group 3/4 classification (subtypes 7 and 4). This finding suggests that methylation-based subtyping can identify a subset of metastatic patients who may benefit from de-escalated, less neurotoxic radiotherapy protocols without compromising survival, a crucial consideration for preserving quality of life. Further refining this concept, a recent integrated risk model for patients with M2/3 (metastatic) medulloblastoma demonstrated that molecular subgrouping (with Group 3 conferring unfavourable outcomes), the pattern of metastatic spread (intracranial disease being more favourable than spinal), and the use of a sandwich chemotherapy strategy (chemotherapy between radiation phases) were all independent prognostic predictors for both event-free and overall survival [43]. This underscores that, for metastatic disease, prognostic accuracy is enhanced by integrating molecular data with detailed clinical, radiologic, and treatment variables.

Beyond DNA methylation, other epigenetic mechanisms, including histone modifications and chromatin remodelling, contribute to therapeutic response in medulloblastoma [44] (Table 2). Alterations in chromatin regulators can influence tumour plasticity and the ability of cancer cells to survive cytotoxic stress. Although these epigenetic features are less frequently assessed in routine clinical practice, they represent an important area of ongoing research, particularly in high-risk tumours where conventional therapies are ineffective [45]. Epigenetic dysregulation may also underlie mechanisms of acquired resistance, highlighting the potential value of longitudinal epigenetic monitoring.

Transcriptomic profiling has further expanded the landscape of predictive biomarkers by capturing gene expression programmes that reflect both intrinsic tumour biology and interactions with the tumour microenvironment (Table 2). Distinct transcriptional signatures have been associated with treatment response across medulloblastoma subgroups, including programmes related to proliferation, stemness, hypoxia, immune modulation, and metabolic adaptation.

**Table 2:** Summary of epigenetic and transcriptomic biomarkers in childhood medulloblastoma.

Biomarker Type	Key Mechanism/Features	Examples/Specific Signatures	Association with Tumour Biology	Predictive Value/Clinical Relevance	Notes/Emerging Applications	Ref.
DNA Methylation	Epigenetic modification of CpG islands; stable and heritable patterns reflect cellular origin and differentiation state	Subgroup-specific methylation profiles (WNT, SHH, Group 3, Group 4); methylation of promoters for cell cycle genes (CDKN2A, RB1), DNA repair genes (MGMT), and neuronal differentiation markers	Distinguishes molecular subgroups and substructures; reflects differentiation state—more differentiated patterns (neuronal gene hypomethylation) correlate with favourable biology	Aberrant hypermethylation of tumour suppressor genes → chemotherapy/radiotherapy resistance; hypomethylation of oncogenic pathways → treatment sensitivity; methylation-based subtypes 4 and 7 within Group 3/4 predict favourable response to reduced-intensity therapy	Incorporated into diagnostic workflows (EPIC arrays, methylation classifiers); guides risk stratification and clinical trial eligibility	[46,47]
Histone Modifications & Chromatin Remodelling	Epigenetic regulation of chromatin structure affects gene accessibility	Alterations in chromatin regulators	Influences Tumour plasticity and survival under cytotoxic stress	May predict response in high-risk or therapy-resistant Tumours	Under investigation; potential target for epigenetic therapies	[48,49]
Transcriptomic Signatures	Gene expression profiling capturing functional programs	Proliferation, stemness, hypoxia, immune modulation, metabolic adaptation; MYC-driven networks in Group 3	Reflects intrinsic Tumour biology and microenvironment interactions	High MYC/stemness expression → poor response and early relapse; differentiation/apoptosis signatures → favourable response	RNA-seq increasingly used to identify predictive signatures	[50,51]
Non-coding RNAs (microRNAs, lncRNAs)	Post-transcriptional gene regulation	Dysregulated miRNAs/lncRNAs affecting DNA repair, drug resistance, and cell survival	Modulates pathways governing therapy response	Altered expression associated with chemotherapy/radiotherapy sensitivity; potential predictive biomarkers	Potential therapeutic targets; clinical validation ongoing	[52,53]
Liquid Biopsy (CSF-based)	Detection of circulating Tumour nucleic acids (DNA/RNA)	Methylated DNA fragments, Tumour-derived RNA transcripts	Reflects Tumour burden and molecular state	Enables longitudinal monitoring of treatment response and early relapse	Minimally invasive; promising for dynamic biomarker assessment	[54,55]

In Group 3 tumours, the elevated expression of MYC-driven transcriptional networks and stem cell–associated genes has been consistently linked to poor response and early relapse [56]. Conversely, expression patterns indicative of neuronal differentiation and apoptotic competence are more commonly observed in tumours that respond favourably to therapy. The finding suggests that methylation-based subtyping can identify a subset of metastatic patients (specifically those belonging to the more favourable subtypes 4 and 7 within the Group 3/4 continuum) who may benefit from de-escalated, less neurotoxic radiotherapy protocols without compromising survival, a crucial consideration for preserving quality of life.

Non-coding RNAs, including microRNAs and long non-coding RNAs, have also emerged as promising transcriptomic biomarkers of treatment response [57]. These molecules play key regulatory roles in post-transcriptional gene control and have been implicated in pathways governing drug resistance, DNA damage repair, and cell survival. Dysregulated expression of specific microRNAs has been associated with altered sensitivity to chemotherapy and radiotherapy, suggesting their potential utility as both predictive biomarkers and therapeutic targets [58]. Although clinical validation remains limited, advances in RNA sequencing and bioinformatic analysis are accelerating the identification of clinically relevant non-coding RNA signatures.

A significant advantage of epigenetic and transcriptomic biomarkers is their potential compatibility with minimally invasive sampling methods. Preliminary studies have demonstrated the feasibility of detecting tumour-derived nucleic acids, including methylated DNA fragments and RNA transcripts, in cerebrospinal fluid (CSF) [59,60]. However, it is not yet established that the molecular profiles in CSF, particularly methylated DNA fragments, are fully concordant with and accurately reflect the complex landscape of the primary tumour tissue. Despite this limitation, the potential of such liquid biopsy-based strategies for longitudinal monitoring remains a highly active area of investigation.

Despite their promise, several challenges must be addressed before epigenetic and transcriptomic biomarkers can be fully integrated into routine clinical care. Technical variability, data complexity, and the need for specialised analytical expertise limit widespread adoption. Moreover, many transcriptomic signatures have been derived from retrospective cohorts and require prospective validation in uniformly treated patient populations.

## 4. Protein and Imaging Biomarkers

Protein- and imaging-based biomarkers play an increasingly important role in evaluating treatment response in paediatric medulloblastoma by providing clinically accessible and non-invasive indicators that complement molecular and genomic profiling [37,61]. Protein biomarkers reflect downstream biological activity of oncogenic pathways and treatment-induced cellular responses, while advanced imaging biomarkers enable real-time assessment of tumour physiology and microenvironmental changes that often precede measurable alterations in tumour size.

At the protein level, several markers have been associated with tumour aggressiveness, treatment sensitivity, and prognosis (Table 3). Overexpression of MYC protein correlates strongly with poor response to conventional therapy and early relapse [62], particularly in Group 3 medulloblastoma, reinforcing its value as both a prognostic and predictive biomarker. Alterations in p53 protein expression, reflective of TP53 mutational status, are linked to impaired DNA damage response and resistance to radiotherapy and chemotherapy [63]. Elevated levels of anti-apoptotic proteins such as surviving and BCL-2 family members have been associated with reduced treatment-induced cell death and inferior clinical outcomes [64]. Conversely, increased expression of markers associated with neuronal differentiation and apoptotic activation has been correlated with improved therapeutic response, suggesting that protein signatures may serve as dynamic indicators of treatment efficacy.

Cerebrospinal fluid (CSF) has emerged as a particularly valuable source of protein-based biomarkers due to its proximity to the tumour and relative accessibility [65]. Tumour-derived proteins, cytokines, and growth factors detected in CSF can reflect tumour burden and biological activity more accurately than peripheral blood markers [66] (Table 3). In parallel, advances in liquid biopsy technologies have enabled the detection of circulating tumour DNA (ctDNA) and tumour-associated proteins in CSF, offering a minimally invasive approach for monitoring treatment response, detecting minimal residual disease, and identifying early relapse before radiographic progression becomes evident [67]. Serial assessment of these biomarkers holds promise for real-time adaptation of therapy, especially in high-risk patients.

**Table 3:** Key Protein and Imaging Biomarkers for Treatment Response in Paediatric Medulloblastoma: Mechanisms and Clinical Applications.

Biomarker Type	Examples/Specific Markers	Biological Basis/Mechanism	Predictive/Prognostic Value	Clinical/Practical Applications	Notes/Emerging Approaches	Ref.
Protein Biomarkers	MYC, p53, survivin, BCL-2, neuronal differentiation markers	Reflect downstream activity of oncogenic pathways, apoptosis regulation, and differentiation	MYC overexpression → poor response and early relapse (esp. Group 3); p53 alterations → impaired DNA repair, therapy resistance; neuronal differentiation markers → improved response	Detection in tumour tissue or CSF; guide risk stratification and therapy adaptation	CSF-derived proteins provide minimally invasive monitoring; serial assessment allows real-time treatment evaluation	[68]
Liquid Biopsy/CSF Protein Markers	Tumour-derived proteins, cytokines, growth factors, ctDNA	Reflect tumour burden and biological activity	Early detection of minimal residual disease, monitoring of treatment response, and prediction of relapse	Minimally invasive; can detect molecular relapse before radiographic progression	Integration with molecular and protein biomarkers enhances longitudinal monitoring	[69]
Diffusion MRI (DWI/ADC)	ADC values from diffusion-weighted imaging	Surrogate of tumour cellularity	Early increases in ADC indicate an effective cytotoxic response	Non-invasive assessment of early treatment response	Functional imaging complements anatomical MRI	[70]
Perfusion MRI	Dynamic susceptibility contrast, arterial spin labelling	Measures tumour vascularity and blood flow	Changes correlate with treatment sensitivity/resistance	Evaluate tumour physiology in real-time	Provides insight into microenvironmental adaptations to therapy	[71]
Magnetic Resonance Spectroscopy (MRS)	Choline, N-acetylaspartate, lactate	Metabolic profiling of tumours	Metabolite shifts indicate therapy response or progression	Complementary to MRI for metabolic assessment	Useful for distinguishing viable tumour vs. post-therapy changes	[72]
PET Imaging	Glucose metabolism tracers, amino acid transport tracers	Functional metabolic imaging	Distinguishes viable tumour tissue from treatment-related changes	Post-therapy evaluation; treatment monitoring	Enhances detection of residual or recurrent disease	[73]
Radiomics & AI-driven Imaging	Quantitative imaging features: shape, texture, heterogeneity, spatial complexity	High-dimensional feature extraction from imaging	Predict molecular subgroup, treatment response, risk of relapse	Non-invasive, longitudinal disease monitoring; supports precision medicine	Integrates with molecular and clinical data for individualised risk prediction	[74]

Imaging biomarkers derived from advanced neuroimaging techniques provide complementary functional information beyond conventional anatomical MRI [75] (Table 3). Diffusion-weighted imaging (DWI) and apparent diffusion coefficient (ADC) measurements serve as surrogate markers of tumour cellularity, with early increases in ADC values often indicating effective treatment-induced cytotoxicity [76,77]. Perfusion MRI techniques, including dynamic susceptibility contrast and arterial spin labelling, enable assessment of tumour vascularity and blood flow, which may change rapidly in response to therapy and correlate with treatment sensitivity or resistance [78].

Magnetic resonance spectroscopy (MRS) offers additional metabolic insight by quantifying tumour-associated metabolites such as choline, N-acetylaspartate, and lactate [79]. Shifts in these metabolic profiles during treatment have been linked to therapeutic response and tumour progression. Functional imaging modalities such as positron emission tomography (PET), using tracers targeting glucose metabolism or amino acid transport, further enhance the ability to distinguish viable tumour tissue from treatment-related changes, particularly in the post-therapy setting [80].

More recently, radiomics and artificial intelligence–driven image analysis have emerged as powerful tools for extracting high-dimensional quantitative features from routine imaging studies [81]. Radiomic signatures capturing tumour shape, texture, heterogeneity, and spatial complexity have demonstrated potential to predict molecular subgroup, treatment response, and risk of relapse [82,83]. When integrated with clinical and molecular data, imaging-based machine learning models offer a non-invasive means of longitudinal disease monitoring and individualised risk prediction.

Despite these advances, several challenges remain in the clinical translation of protein and imaging biomarkers. Variability in assay platforms, imaging protocols, and analytical methodologies limits reproducibility across centres. Moreover, many biomarkers have been validated retrospectively, highlighting the need for prospective studies incorporating standardised biomarker endpoints [84]. Nevertheless, the integration of protein-based markers, liquid biopsy approaches, and advanced imaging biomarkers represents a critical step toward dynamic, response-adaptive treatment strategies in paediatric medulloblastoma, enabling earlier identification of therapeutic failure and more precise tailoring of therapy to individual patients.

## 5. Challenges, Limitations, Ethical Issues and Future Directions

Despite progress in biomarker discovery, several obstacles limit clinical translation. Key barriers include biological heterogeneity within and between molecular subgroups; intra-tumoral heterogeneity and clonal evolution under therapeutic pressure can cause discordance between diagnostic and relapse biomarkers [85,86]. Biomarkers from single tumour regions or baseline samples may incompletely capture dynamic disease biology, limiting predictive reliability over time [87].

Another major limitation is the restricted availability of high-quality tumour material, particularly in paediatric patients, where surgical sampling must prioritise neurological safety and long-term function [88]. Small tissue volumes often preclude comprehensive multi-omics analyses, and repeat biopsies are rarely feasible, especially at relapse. This constraint not only limits biomarker discovery but also raises ethical concerns regarding the balance between scientific value and procedural risk in children [89,90]. While liquid biopsy approaches using cerebrospinal fluid or blood offer promising, less invasive alternatives, their sensitivity, specificity, and technical reproducibility remain variable, and standardised protocols for clinical implementation are still lacking.

Technical and methodological heterogeneity represents an additional challenge with ethical implications for equity and reproducibility [91]. Differences in sequencing platforms, bioinformatic pipelines, protein detection assays, and imaging acquisition parameters across institutions introduce variability that undermines cross-study comparability and may lead to inconsistent clinical decision-making [92]. Imaging biomarkers and artificial intelligence–based models are particularly susceptible to protocol-dependent variation, overfitting, and limited generalisability, raising concerns about transparency, interpretability, and unintended bias [93]. Without rigorous external validation, the deployment of such tools risks reinforcing health inequities or exposing patients to inappropriate treatment recommendations.

**Figure 1:**
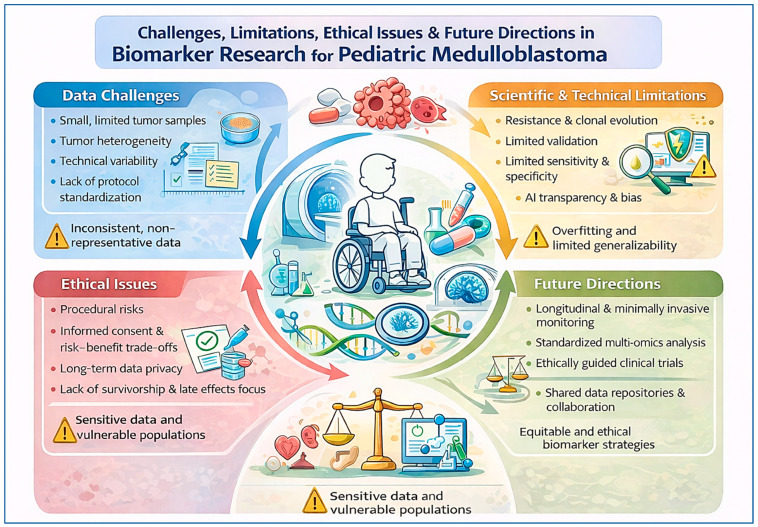
An outline of current challenges, limitations, ethical issues and development prospects. The ChatGPT-4 version (OpenAI, San Francisco, CA, USA) AI tool was used to generate the Figure.

Ethical considerations are especially salient in the context of biomarker-driven clinical trials [94] (Figure 1). Although molecular subgrouping is now routinely incorporated into risk stratification, most additional biomarkers of treatment response have not yet been prospectively validated as decision-making tools in randomised trials [95,96]. Treatment de-escalation based on favourable biomarkers carries the ethical risk of undertreatment and disease relapse, whereas treatment intensification in biomarker-defined high-risk groups may exacerbate toxicity without guaranteed benefit [97]. In paediatric oncology, where patients cannot provide fully informed consent and families must navigate complex risk–benefit trade-offs, the use of unvalidated biomarkers demands particular caution and transparent communication.

Data governance and privacy also represent growing ethical challenges as biomarker research increasingly relies on large-scale genomic, epigenomic, and imaging datasets [98] (Figure 1). The collection, storage, and sharing of sensitive molecular data from children raise important questions regarding consent, data ownership, long-term use, and the potential for re-identification [99]. Ensuring robust safeguards, clear consent frameworks, and responsible data-sharing practices is essential to maintaining public trust and protecting patient rights.

Importantly, current biomarker research has focused predominantly on predicting oncologic outcomes, with comparatively limited attention to biomarkers of treatment-related toxicity and long-term survivorship [100]. In paediatric medulloblastoma, where cure is frequently accompanied by significant neurocognitive, endocrine, auditory, and psychosocial sequelae, the absence of validated biomarkers predicting late effects represents both a scientific and ethical gap [101]. Without such markers, precision medicine risks prioritising survival alone, rather than supporting holistic, life-course–oriented care that reflects the long-term interests of survivors.

Looking ahead, future directions in this field will require a shift towards integrative, longitudinal, and ethically grounded biomarker strategies. Advances in multi-omics technologies, single-cell and spatial profiling, and artificial intelligence–driven data integration offer opportunities to capture tumour complexity and temporal evolution better, while minimally invasive monitoring approaches may reduce procedural burden. Equally important will be the development of standardised analytical frameworks, shared data repositories, and prospective biomarker-driven clinical trials with long-term follow-up that explicitly incorporate ethical oversight, patient-reported outcomes, and survivorship endpoints. Successful translation will depend on close collaboration among clinicians, researchers, regulatory authorities, ethicists, and patient advocates to ensure that biomarker innovations are not only scientifically robust but also ethically sound, equitable, and clinically meaningful. Addressing these challenges is essential to fully realise the promise of biomarker-guided precision therapy and to improve both survival and quality of life for children with medulloblastoma.

## 6. Conclusions

The integration of biomarkers into the management of paediatric medulloblastoma has transformed both prognostic assessment and therapeutic decision-making. Molecular subgrouping, epigenetic and transcriptomic profiling, protein markers, and advanced imaging techniques collectively provide a multidimensional understanding of tumour biology and treatment response. These biomarkers enable more accurate risk stratification, inform therapy de-escalation or intensification, and support the development of novel targeted treatments. However, challenges, including intra-tumour heterogeneity, limited prospective validation, and ethical considerations in paediatric populations, must be addressed to realise their full clinical potential. Future research should focus on longitudinal, multi-omics, and minimally invasive biomarker strategies, coupled with standardised protocols and patient-centred outcomes, to ensure that biomarker-guided approaches improve both survival and long-term quality of life for children with medulloblastoma.

## Data Availability

No new data were created or analyzed in this study.
